# Hepatic regeneration following radiation-induced liver injury is associated with increased hepatobiliary secretion measured by PET in Göttingen minipigs

**DOI:** 10.1038/s41598-020-67609-y

**Published:** 2020-07-02

**Authors:** Kristoffer Kjærgaard, Britta Weber, Aage Kristian Olsen Alstrup, Jørgen Breede Baltzer Petersen, Rune Hansen, Stephen Jacques Hamilton-Dutoit, Frank Viborg Mortensen, Michael Sørensen

**Affiliations:** 1 Department of Nuclear Medicine and PET Centre, Aarhus University Hospital, 8200 Aarhus N, Denmark; 2 Department of Hepatology and Gastroenterology, Aarhus University Hospital, 8200 Aarhus N, Denmark; 3 Department of Oncology, Aarhus University Hospital, 8200 Aarhus N, Denmark; 4 Department of Pathology, Aarhus University Hospital, 8200 Aarhus N, Denmark; 5 Department of Surgery, Aarhus University Hospital, 8200 Aarhus N, Denmark

**Keywords:** Physiology, Gastroenterology, Molecular medicine

## Abstract

Normal liver tissue is highly vulnerable towards irradiation, which remains a challenge in radiotherapy of hepatic tumours. Here, we examined the effects of radiation-induced liver injury on two specific liver functions and hepatocellular regeneration in a minipig model. Five Göttingen minipigs were exposed to whole-liver stereotactic body radiation therapy (SBRT) in one fraction (14 Gy) and examined 4–5 weeks after; five pigs were used as controls. All pigs underwent in vivo positron emission tomography (PET) studies of the liver using the conjugated bile acid tracer [*N*-methyl-^11^C]cholylsarcosine ([^11^C]CSar) and the galactose-analogue tracer [^18^F]fluoro-2-deoxy-d-galactose ([^18^F]FDGal). Liver tissue samples were evaluated histopathologically and by immunohistochemical assessment of hepatocellular mitosis, proliferation and apoptosis. Compared with controls, both the rate constant for secretion of [^11^C]CSar from hepatocytes into intrahepatic bile ducts as well as back into blood were doubled in irradiated pigs, which resulted in reduced residence time of [^11^C]CSar inside the hepatocytes. Also, the hepatic systemic clearance of [^18^F]FDGal in irradiated pigs was slightly increased, and hepatocellular regeneration was increased by a threefold. In conclusion, parenchymal injury and increased regeneration after whole-liver irradiation was associated with enhanced hepatobiliary secretion of bile acids. Whole-liver SBRT in minipigs ultimately represents a potential large animal model of radiation-induced liver injury and for testing of normal tissue protection methods.

## Introduction

Positron emission tomography (PET) has shown great potential for evaluating liver-specific functions in vivo*,* both regionally and for the whole liver^1^. Excretion of bile acids and galactose metabolism are such liver-specific functions^2,3^ that can be quantified using computed positron emission tomography (PET/CT) with the radio-labelled conjugated bile acid [*N*-methyl-^11^C]cholylsarcosine ([^11^C]CSar)^4,5^ and the galactose analogue [^18^F]fluoro-2-deoxy-d-galactose ([^18^F]FDGal)^6,7^, respectively.

Before use in humans, novel PET/CT methods assessing liver functions are validated in large animal models such as the pig, with blood sampling from the portal vein and direct measurement of hepatic blood flow^8,9^. Because of the limited number of large animal models of parenchymal liver injury and fibrosis^10–12^, PET tracers have so far only been tested in healthy pigs.

Stereotactic body radiation therapy (SBRT) is increasingly being used to deliver high doses of radiation to hepatic malignancies with precision^13^. The liver parenchyma, however, is sensitive to irradiation^14,15^, and high doses of hepatic irradiation induce both acute and chronic injuries^16^. In a pilot study, we used SBRT to deliver a uniform radiation dose of 14 Gy to the whole liver in Göttingen minipigs, after which they developed significant histological liver injury when examined 4–5 weeks post-treatment (M. Sørensen, unpublished data). This method represents a potential new large animal model for radiation-induced liver injury.

The aim of this study was to examine the effects of SBRT-induced liver injury on hepatobiliary and hepatocytosolic liver function in a minipig model measured by invasive [^11^C]CSar and [^18^F]FDGal PET/CT, respectively. Parenchymal injury and hepatocellular regeneration were characterized by histopathological evaluation and immunohistochemical analysis.

## Results

All pigs exposed to SBRT survived to undergo PET studies. One irradiated pig (R2) developed local radiation-induced gastritis. The hepatic venous catheter had retracted during the PET studies in one control pig (C2) and as a result, this pig was not included in the analysis of [^11^C]CSar data. When compared with baseline, blood levels of aspartate aminotransferase (AST) and the aspartate aminotransferase/alanine aminotransferase (AST/ALT) ratio were significantly increased in irradiated pigs at the time of PET studies, while platelets were reduced. The remaining blood tests were unaffected (Table 1), and there were no significant differences between baseline blood tests from irradiated pigs and control pigs (data not shown).

**Table 1:** Biochemical blood test values from irradiated pigs at baseline and after irradiation: data are shown as mean (95% confidence interval) or median [range]; the groups were compared using the Paired *t* test or Wilcoxon signed-rank test, when appropriate (n = 5 per group).

	Baseline	PET studies
ALT	64.2 (48.3; 80.1)	57.8 (39.6; 76.0)
AST	47.5 [32.0–53.0]	109.0* [66.0–314.0]
AST/ALT ratio	0.82 [0.45–1.04]	1.94* [1.48–4.55]
GGT	84 (62.2; 105.8)	99.8 (77.4; 122.2)
Bilirubin	< 5	< 5
ALP	104.2 (66.9; 141.5)	97.8 (70.6; 125.0)
Albumin	17.8 (16.2; 19.4)	17.2 (14.2; 20.2)
Amylase	2,433 [1608–2977]	2,774 [1095–3666]
INR	1.10 [1.00–1.20]	1.10 [1.00–1.30]
Platelets	510 (388; 631)	364* (296; 433)

*ALT* alanine aminotransferase (units/L plasma), *AST* aspartate aminotransferase (units/L plasma), *GGT* gamma-glutamyl transferase (units/L plasma), bilirubin (µmol/L plasma), *ALP* alkaline phosphatase (units/L plasma), albumin (g/L plasma), *INR* International Normalized Ratio (plasma), platelets (× 10^9^/L blood).

*P < 0.05 when compared with baseline.

### Hepatobiliary function ([^11^C]CSar PET)

Kinetic clearance parameters calculated from blood measurements of [^11^C]CSar did not show any overall difference in hepatobiliary function between irradiated pigs and control pigs. That is, with regard to uptake across the hepatocyte membrane (*PS*_mem_, *P* > 0.3), and to both flow-independent and flow-dependent clearances of bile acids (*Cl*_int_ and *Cl*_sys_, both *P* > 0.3).

**Figure 1:**
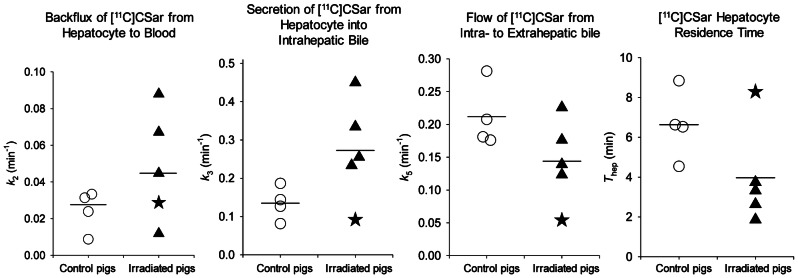
Individual rate constants for the hepatobiliary transport of [^11^C]CSar estimated from PET in control and irradiated pigs. Control pigs are shown as white circles, irradiated pigs as black triangles (pig with inflammatory cholestasis indicated as a star; Pig R4). Horizontal bars represent group means.

**Figure 2:**
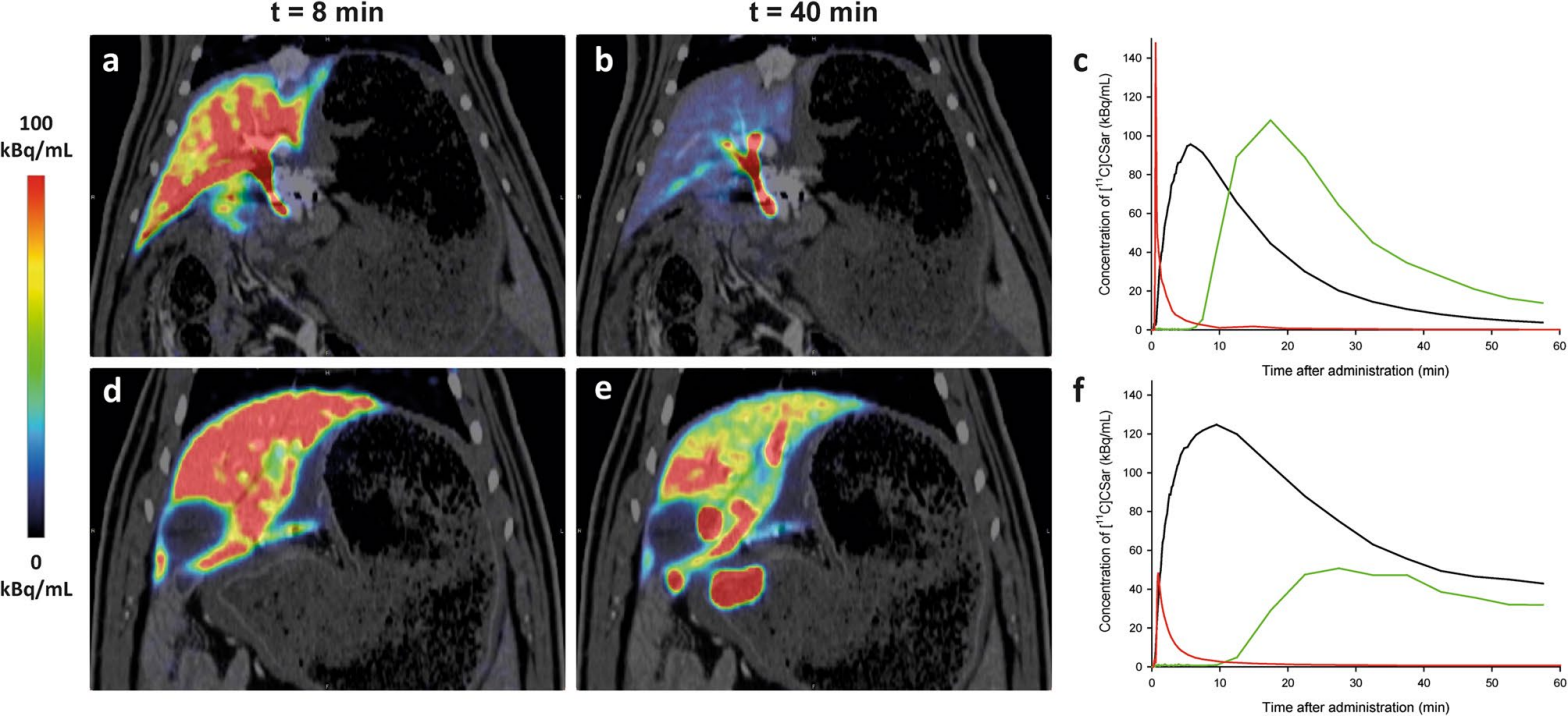
PET/CT images and time activity curves from dynamic [^11^C]CSar scans in a control pig (Pig C3; **a**–**c**) and an irradiated pig with lobular inflammation and impaired hepatobiliary function (Pig R4; **d**–**f**). The PET/CT images are coronal and show average concentration recorded 7.5–8.5 min (**a**, **d**) and 37.5–42.5 min (**b**,**e**) following bolus injection of 78 MBq [^11^C]CSar. Panels (**c**,**f**) display the time course of [^11^C]CSar concentration in arterial blood (red), liver tissue (black), and the common hepatic duct (CHD), divided by 50 (green). In both pigs, [^11^C]CSar is rapidly cleared from blood and accumulated in the liver tissue. In the control pig, [^11^C]CSar is almost completely cleared from the liver and excreted into the CHD at 37.5–42.5 min, while high concentrations of [^11^C]CSar still remain in the liver of the irradiated pig and is excreted at a reduced rate.

The flow-dependent unidirectional clearance of [^11^C]CSar from blood to hepatocytes was unaffected in irradiated pigs (*K*_1_, *P* > 0.3). The rate constants for secretion of [^11^C]CSar from hepatocytes to intrahepatic bile and backflux from hepatocytes to blood were increased in three irradiated pigs, but the mean group increase was not significant (*k*_3_, *P* = 0.09; *k*_2_, *P* = 0.18). The mean hepatocyte residence time of [^11^C]CSar was reduced from 6.6 min in the control pigs to 4 min in the irradiated pigs (*T*_hep_, *P* = 0.12), and the rate constant for flow of [^11^C]CSar in bile out of the liver was also reduced (*k*_5_, *P* = 0.12). The individual kinetic parameters are given in Fig. 1. For the two irradiated pigs with histological liver injury, i.e. fibrosis and lobular inflammation (R1 and R4; see below), secretion of [^11^C]CSar out of the hepatocyte was not increased, but rather impaired for the one (R4; Fig. 2) when compared with the control group. Excluding the pig with radiation-induced cholestasis (R4) from the statistical analysis, the hepatobiliary secretion and overall transport out of the hepatocyte were significantly increased for the irradiated pigs (*k*_3_, *P* = 0.04; *T*_hep_, *P* = 0.003).

### Hepatocytosolic function ([^18^F]FDGal PET)

**Figure 3:**
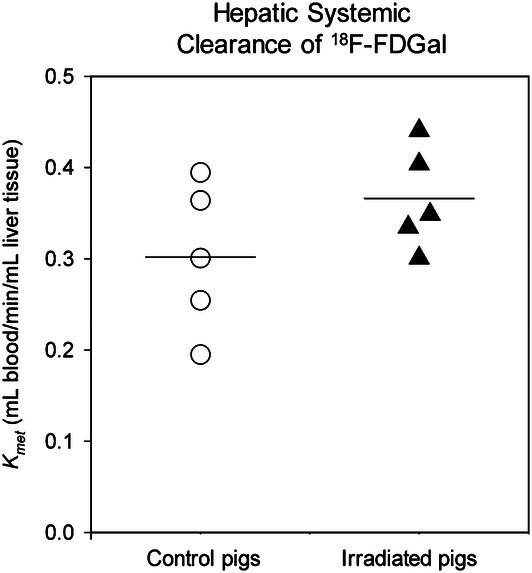
Individual clearance values of [^18^F]FDGal PET in control pigs and irradiated pigs. Control pigs are shown as white circles and radiation pigs as black triangles. Horizontal bars represent groups means.

As shown in Fig. 3, the mean hepatic systemic clearance of [^18^F]FDGal in irradiated pigs was slightly increased, but not significantly different when compared with control pigs (0.37 mL blood/min/mL liver tissue vs. 0.30 mL blood/min/mL liver tissue, *P* = 0.18).

### Histopathology and immunohistochemistry

**Figure 4:**
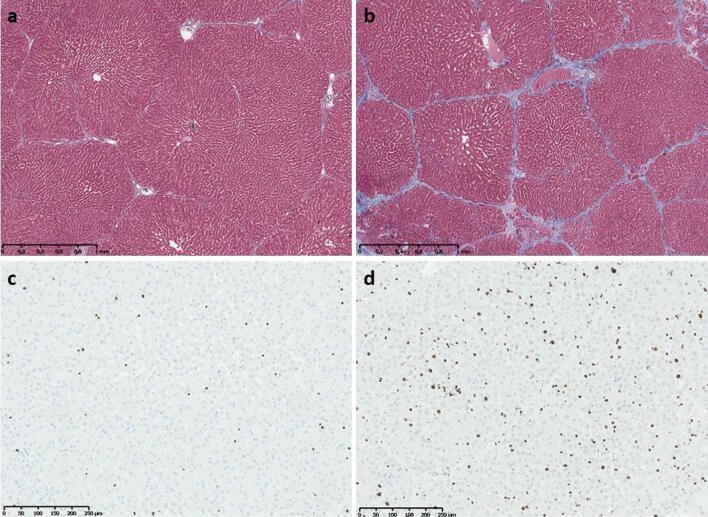
Histological images from a control pig (Pig C4; **a**,**c**) and an irradiated pig with hepatic fibrosis (Pig R1; **b**,**d**). (**a**,**b**) Tissue samples sections stained with Masson’s trichrome stain for evaluation of hepatic fibrosis in a control pig (**a**) and an irradiated pig (**b**) with mild fibrosis. (**c**,**d**) Tissue sections immunohistochemically stained with an antibody against the nuclear antigen Ki-67, specific for hepatocytes in the proliferating phases of the cell cycle. When compared with the control pig, the number of proliferating hepatocytes in the irradiated pig (**d**) is markedly higher.

**Figure 5:**
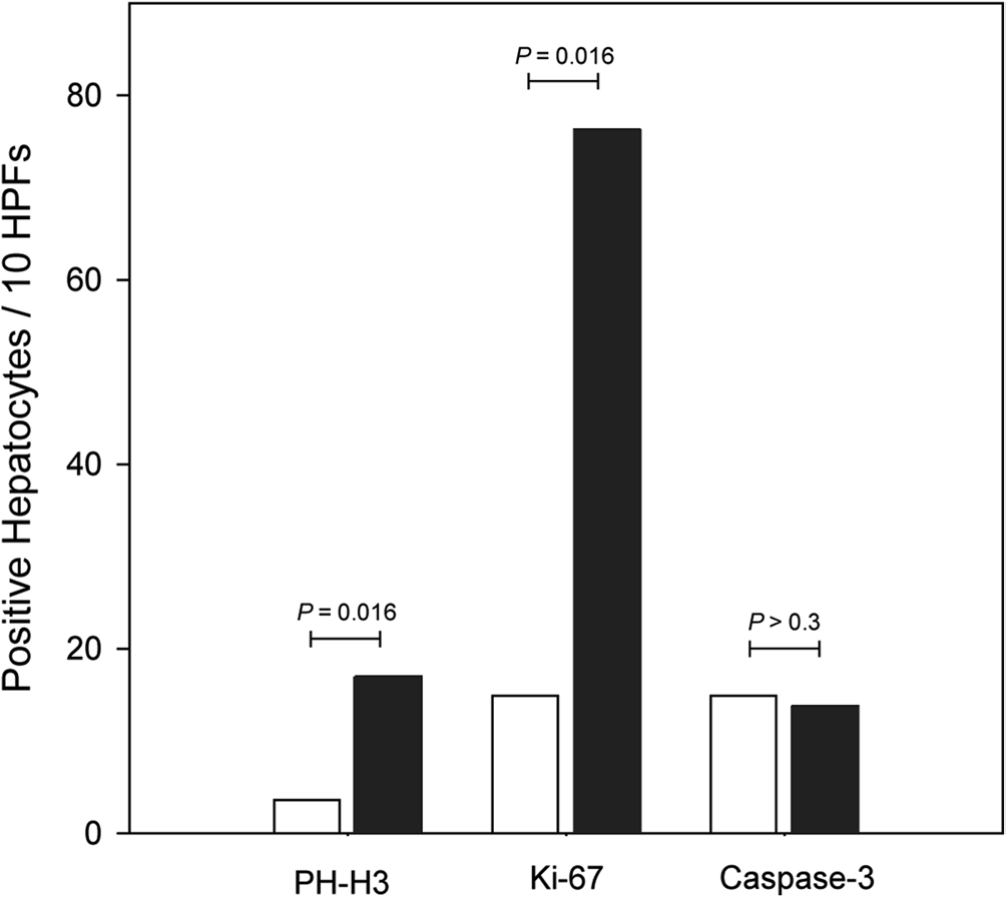
Hepatocellular mitosis (PH-H3), proliferation (Ki-67) and apoptosis (caspase-3) in hepatic tissue of control and irradiated pigs. The activity was estimated by counting the number of positive hepatocytes per 10 high-power-fields (HFPs). White bars are control pigs, black bars are irradiated pigs. Values are displayed as group medians; the groups were compared using the Wilcoxon rank sum test (n = 5 per group).

One pig (R1) developed mild hepatic fibrosis (Fig. 4), and another pig (R4) showed mild lobular inflammation after SBRT. Histological features in liver tissue sections from the remaining irradiated and control pigs were normal with regard to fibrosis, steatosis and lobular inflammation. However, when compared with controls, immunohistochemically stained sections from irradiated pigs contained a significantly higher number of hepatocytes undergoing both mitosis (PH-H3; median 17.0 positive hepatocytes per 10 HPFs vs. 3.6 positive hepatocytes per 10 HPFs; *P* = 0.016) and proliferation (Ki-67; median 76.3 positive hepatocytes per 10 HPFs vs. 12.8 positive hepatocytes per 10 HPFs; *P* = 0.016) (Fig. 5). There was no difference in the number of hepatocytes undergoing apoptosis in the two groups (caspase-3; median 13.8 positive hepatocytes per 10 HPFs vs. 14.9 positive hepatocytes per 10 HPFs; *P* > 0.3). In addition, we found a highly significant correlation between proliferative activity estimated from Ki-67 staining and AST levels in blood on the day of liver PET studies (R^2^ = 0.970, *P* < 0.0001) for all pigs.

## Discussion

In this experimental animal study, we examined the effects of radiation-induced liver injury on PET-measured hepatobiliary and hepatocytosolic function by invasive [^11^C]CSar and [^18^F]FDGal PET/CT in Göttingen minipigs. Interestingly, the mean rate constants for secretion of [^11^C]CSar from hepatocyte to bile and for backflux to blood were both doubled in irradiated pigs when compared with control pigs, while the mean rate constant for bile flow was two-thirds of that in control pigs. The hepatocytosolic function measured as the hepatic systemic clearance of [^18^F]FDGal was slightly increased in irradiated pigs.

The accuracy of the SBRT allowed us to distribute a homogenous radiation dose with a planning target volume coverage of > 95% to the whole liver, which was crucial for elimination of functional and regenerative compensation in any nonirradiated liver tissue^17^. We chose the Göttingen minipig since it does not grow substantially over time from exposure to examination^18^. Even so, the individual tolerance towards hepatic irradiation varied considerably, as shown by the large differences in liver function and histopathology observed in irradiated pigs. Such variation in radiation tolerance is also observed in patients recovering from hepatic radiotherapy^19^.

The observed differences in the studied liver functions did not reach statistical significance. This could likely be ascribed to individual radiation tolerance, as evident in one pig (R4) where irradiation caused lobular inflammation and severe cholestasis; this was in contrast to the remaining irradiated pigs, where the histological interpretation was more in line with a state of recovery. Added the variation in radiation tolerance, a limitation to our study design was that the invasiveness of the PET methods excludes the possibility of paired analysis. Although a tendency was seen in this study that whole-liver irradiation affected the hepatobiliary excretion, the combination of one outlier pig and overestimated power resulted in only border-significant findings.

The observed individual changes do however fit with what has previously been observed in patients. The liver of irradiated pigs seems in a recovering state 4–5 weeks after SBRT with five-fold increases in hepatocellular mitosis and proliferation associated with increased secretion of [^11^C]CSar from the hepatocytes; this compares with observations from an [^11^C]CSar PET case study of a patient with drug-induced liver injury who, six months into recovery, also displayed a significant increase in hepatobiliary secretion when compared with the [^11^C]CSar PET scan performed during the acute phase^20^, and with values from healthy subjects^5^. In addition, the impaired secretion of bile and bile flow observed in the pig with lobular inflammation is in alignment with the changes observed in patients with acute inflammation of the liver^5^. Overall, the present results from this invasive pig study validates the ability of the applied PET tracers to detect minor inter-individual changes, even in the absence of histological alterations, thus underlining the sensitivity and power of functional PET when studying liver injury in patients. The increased hepatocellular proliferation in the irradiated pigs may also explain the slight increase in clearance of [^18^F]FDGal, as hepatic regeneration has been associated with accelerated galactose metabolism in previous studies in rats and humans^21,22^. The compensatory increase in liver functions subsequent to acute injury revealed in the present study is a novel finding, and it would be interesting for future studies to shed light on the phenomenon, especially in patients who exhibit parenchymal liver injury after irradiation.

In rats, high doses of radiation to the liver in a single fraction (15, 20, 30 Gy) have been shown to induce hepatic fibrosis and even cirrhosis^16,23^, resulting in distinct increases in AST, alkaline phosphatase and bilirubin. Notably, 30 Gy caused death for more than half the rats in the study^23^. We observed increased AST-levels as well, highest for the pig that developed fibrosis (R1). The uniform 14 Gy dose of whole-liver irradiation at a single fraction did not induce consistent hepatic fibrosis or chronic liver damage as was seen in our pilot studies, but did cause hepatic fibrosis in one pig (R1) and lobular inflammation in another (R4). Considering the welfare of the animals, we did not increase radiation dose from that used in our pilot studies, though we hypothesize that increased doses would induce consistent chronic liver injury as well as functional impairments similar to that observed in pig R4.

As various radiation-based treatments for hepatic malignancies become increasingly relevant^24^, several studies have investigated new methods to spare surrounding healthy liver tissue for reduction of the feared complications associated with radiotherapy^25^. We show for the first time that SBRT is a useful method to produce large animal models of radiation-induced liver injury, in which dosage and fractionation as well as the progressive hepatic injury following radiotherapy, can be studied invasively. Importantly, the model allows for studies of radiotherapeutic intervention on normal hepatic tissue and liver-specific PET/CT methods in a highly translational, preclinical setting.

In summary, uniform whole-liver irradiation in Göttingen minipigs induced an upregulation of studied liver functions, doubling the transport of [^11^C]CSar out of hepatocytes and slightly increasing the hepatic systemic clearance of [^18^F]FDGal as compared with control pigs. However, statistical significance was hindered by the small sample size. The enhancement of liver functions were accompanied by a consistent and distinct increase in hepatocellular regeneration 4–5 weeks following radiation, even in the absence of histopathological damage. Functional bile acid PET using [^11^C]CSar identified small changes in the separate steps of hepatobiliary excretion that were otherwise not revealed by blood clearance measurements.

## Methods

### Study design

We used ten 12-month old female Göttingen minipigs (mean body weight 28.9 kg; range 26.0–32.0) from Ellegaard Minipigs (Dalmose, Denmark). Five pigs chosen by randomization were anesthetized and exposed to a uniform dose (14 Gy) of whole-liver irradiation using SBRT, observed for 4–5 weeks, then re-anesthetized, surgically prepared (see below) and examined by a 60-min dynamic [^11^C]CSar PET scan followed by a 20-min dynamic [^18^F]FDGal PET scan; [^18^F]FDGal PET was initiated at least 100 min after injection of [^11^C]CSar to ensure full radioactive decay of the [^11^C] isotope. After PET/CT, the pigs were euthanized, and the liver was removed. Liver tissue samples were obtained for histopathological evaluation and immunohistochemical analysis. Five control pigs were not subjected to SBRT but underwent PET/CT studies and evaluation of tissue samples.

### Animals

The protocol was approved by The Danish Animal Experiments Inspectorate (2017-15-0201-01277), and all experiments were performed according to the current law on animal experimentation and ethics. The environmental conditions were 20 °C and 50-55% relative humidity, and the air was changed 8 times every hour, and with 12:12 hours of light and darkness. They were fed a restricted pellet diet with free access to tap water. The pigs were clinically healthy before studies, and the well-being of the animals was monitored twice per day during follow-up.

### Anaesthesia

The animals were sedated and anesthetized with an infusion of midazolam, *S*-ketamine and propofol, mechanically ventilated, and kept physiologically stable (placed on a thermostatically controlled heating blanket, keeping the rectal temperature between 38.5 and 39.5 °C). Before CT-guided SBRT, a gastric feeding tube was placed to eliminate excessive air in the stomach.

### Stereotactic body radiation therapy (SBRT)

A CT scan (Siemens Biograph 64 Truepoint, 300 effective mAs, 120 kV, pitch 0.8, slice thickness 2 mm) was performed using intravenous contrast media (Visipaque 320 mg I/mL; 2 mL/kg body weight; 2 mL/min) with imaging of the late portal phase (40 s delay) for plotting of the liver and adjacent risk organs. The liver was contoured as target volume; the stomach, spinal cord and intestines were contoured as the most important organs at risk. The planning target volume was defined as the whole liver with a margin of 1 cm. A subsequent treatment plan distributing a uniform dose of 14 Gy with a PTV coverage of > 95% was constructed using the Eclipse treatment planning system (Varian, Palo Alto, CA). The pig was then moved to the therapy bed, and a cone-beam CT scan was performed for verification of the liver’s position. Radiation was delivered in one fraction in an accelerator (Varian, Trilogy). During the planning CT and radiotherapy, ventilation was paused momentarily to discontinue respiratory motion of the liver.

### Surgical preparation

Before PET studies, the liver was accessed through an abdominal midline incision. For measurement of hepatic blood flow, ultrasound transit-time flow meter probes (VeriQ; Medistim, Denmark) were placed around the hepatic artery (*F*_HA_, mL blood/min) and portal vein (*F*_PV_, mL blood/min). Total liver blood flow (*F*, mL blood/min) was calculated as the sum of *F*_HA_ and *F*_PV_. Hepatic blood perfusion, *Q* (mL blood/min/mL liver tissue), was calculated as *Q* = *F*/*V*, where *V* (mL liver tissue) is the liver volume, calculated as the liver weight corrected for a tissue density of 1.07 g/mL liver tissue^26^. For intravenous administration of tracer, a catheter was inserted in the femoral vein. For blood sampling, catheters were placed directly in the femoral artery and portal vein, and in a hepatic vein via the right jugular vein. Surgical procedures were followed by one hour of physiological stabilization before PET studies.

### PET studies

The pig was placed in supine position in a Siemens Biograph 64 Truepoint PET/CT camera. A low-dose CT scan (50 effective mAs, 120 kV, pitch 0.8, slice thickness 5 mm) was performed before each PET scan for attenuation correction of emission data and anatomical co-registration of PET data. Concentrations of [^11^C]CSar and [^18^F]FDGal were measured in blood samples (see below) using a well counter (Packard), and time courses for the concentration in blood were generated (kBq/mL blood vs. min). PET measurements and blood concentrations were cross-calibrated with the PET-camera and corrected for radioactive decay back to start of the PET scan.

#### *[*^*11*^*C]CSar PET*

A median dose of 78 (range 46–110) MBq [^11^C]CSar, produced in-house^27^, was administered as an intravenous bolus during the initial 20 s of a 60-min PET scan. PET data were reconstructed using attenuation weighted ordered subset expectation maximization with resolution recovery (TrueX 3D) with four iterations, 21 subsets, a 336 × 336 × 109 matrix and a 2 mm Gaussian filter. Final PET image voxel size was 2 × 2 × 2 mm^3^; time frame structure was 18 × 5 s, 15 × 10 s, 4 × 30 s, 4 × 60 s, 10 × 300 s.

During the PET scan, successive blood samples were collected from a femoral artery *C*_A_(*t*), the portal vein, *C*_PV_(*t*), and a hepatic vein, *C*_out_(*t*). The time course of the flow-weighed mixed input of tracer to the liver from the hepatic artery and portal vein, *C*_in_(*t*) (kBq/mL blood vs. min), was calculated as:

$${C}_{in}\left(t\right)={F}_{HA}{C}_{HA}\left(t\right)+\left(1-{F}_{HA}\right){C}_{PV}(t)$$

Because of surgical difficulties, we were unable to sample blood from the portal vein in five pigs (three irradiated pigs, two controls). In these cases, *C*_PV_(*t*) was estimated using a model for transfer of tracer from the artery to the portal vein through the prehepatic splanchnic bed^28^:

$${C}_{PV}\left(t\right)={\int }_{0}^{\infty }h\left(t-\tau \right){C}_{A}\left(\tau \right) d\tau$$

The model includes measured *C*_A_(*t*) and time, τ, which is determined by a tracer-specific parameter, β:

$$h\left(t\right)=\frac{\beta }{{\left(t+\beta \right)}^{2}}$$

We used a mean value of β determined from the experiments with portal vein sampling. The model-derived *C*_PV_(*t*)*,* estimated using a mean value of β, was almost identical to the measured blood data, when tested.

#### *[*^*18*^*F]FDGal PET*

A median dose of 83 (range 70–91) MBq [^18^F]FDGal, produced in-house^29^, was administered as an intravenous bolus during the initial 20 s of a 20-min PET scan. PET data were reconstructed using iterative processing with four iterations, 21 subsets, 168 matrices and a 2 mm Gaussian filter. Time frame structure was 20 × 5 s, 1 × 10 s, 3 × 20 s, 1 × 30 s, 1 × 40 s, 2 × 60 s, 7 × 120 s.

During the PET scan, successive blood samples were collected from the femoral artery, *C*_A_(*t*)^30^.

### Histopathology and immunohistochemistry

Immediately after removal and weighing of the liver, multiple tissue samples were obtained from all liver lobes, fixed in buffered formalin for 24 h, and cut into 2 mm thick parallel blocks using a tissue slicer. These were processed and embedded in paraffin by standard protocols. Using routine techniques, tissue sections were cut and stained with haematoxylin and eosin (H&E) for standard histology and Masson’s trichrome (MT) for assessment of fibrosis. Hepatocellular mitotic activity and proliferation were assessed by immunohistochemistry using primary antibodies against phosphohistone H3 (anti-PHH3; ready to use rabbit polyclonal antibody; Cell Marque, Rocklin, CA), Ki-67 (anti-Ki-67; ready to use rabbit monoclonal antibody; Ventana Medical Systems, Tucson, AZ), and caspase-3 (anti-caspase-3; rabbit monoclonal antibody (5A1E), diluted 1:2000; Cell Signaling, Danvers, MA), respectively. All staining procedures were performed on an automated slide staining system (BenchMark Ultra, Ventana Medical Systems) using OptiView Detection Kit (Ventana Medical Systems) for signal detection. Evaluation of the histological and immunohistochemical features was performed by microscope (Nikon Eclipse 80i microscope, × 10 eyepiece, × 40 objective). Hepatocellular mitotic activity, proliferation and apoptosis were evaluated semi-quantitatively by counting positive hepatocytes per 10 high-power fields (HPF = 0.57 mm). An experienced (blinded) hepatopathologist (SD) analysed all sections.

### Analysis of PET and blood data

PET images were analysed using the PMOD software version 3.7 (PMOD Technologies Ltd, Zürich, Switzerland). Using combined PET/CT images, a volume-of-interest (VOI) was drawn in liver tissue, excluding large blood vessels and visible intrahepatic bile ducts. The VOIs were used to generate the time courses of liver tissue concentration of [^11^C]CSar and [^18^F]FDGal, *C*_liver_*(t)* (kBq/cm^3^ liver tissue), for kinetic analysis of PET data. Mean VOI volumes for [^11^C]CSar and [^18^F]FDGal PET were 21.5 mL (range 11.5–52.6) and 29.8 mL (range 24.4–34.4) liver tissue, respectively.

#### Analysis [^11^C]CSar PET data

The time course of the hepatic extraction of [^11^C]CSar from blood, *E*(*t*), was calculated from *C*_i_(*t*) and *C*_out_(*t*):

$$E\left(t\right)=\frac{{C}_{in}\left(t\right)-{C}_{out}(t+T)}{{C}_{in}\left(t\right)}$$

The concentration of [^11^C]CSar in *C*_out_(*t*) was corrected for non-steady state by using individual transit times, *T* = *V*_blood_/*Q*, estimated from individually measured hepatic perfusion, *Q*, and a mean fractional blood volume in the liver, *V*_blood_ (0.25 mL blood/mL liver tissue)^31,32^. The time course, *E*(*t*)*,* was used to calculate the unidirectional extraction fraction of [^11^C]CSar from blood to hepatocytes, *E*_0_, and *E*_AUC_ from 0 to 50 min, which approximates steady state hepatic extraction fraction^5^.

The flow-independent permeability-surface area product of the hepatocyte plasma membrane for [^11^C]CSar, *PS*_mem_ (mL blood/min/mL liver tissue), was calculated as^33^:

$$P{S}_{mem}=-Qln(1-{E}_{0})$$

The flow-independent hepatic intrinsic clearance of [^11^C]CSar from blood to bile, *Cl*_int_ (mL blood/min/mL liver tissue), was calculated as^33^:

$$C{l}_{int}=-Qln(1-{E}_{AUC})$$

The flow-dependent hepatic systemic clearance of [^11^C]CSar from blood to bile, *Cl*_sys_ (mL blood/min/mL liver tissue), was calculated as^33^:

$$C{l}_{sys}=Q{E}_{AUC}$$

**Figure 6:**
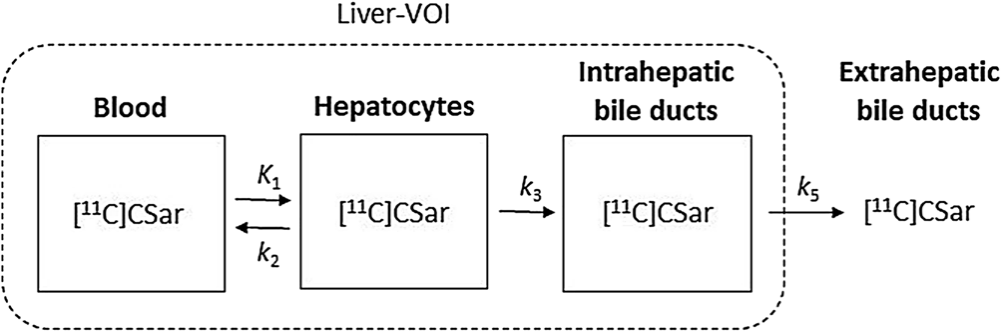
Kinetic two-tissue compartmental model of transport of [^11^C]CSar from blood to bile. *K*_1_: unidirectional clearance from blood to hepatocytes (mL blood/min/mL liver tissue); *k*_2_: rate constant for backflux from hepatocytes to blood (/min); *k*_3_: rate constant for secretion from hepatocytes to intrahepatic bile ducts (/min); *k*_5_: rate constant for bile flow out of the liver (/min). *Liver-VOI* liver volume-of-interest (VOI).

PET data were analysed using a kinetic compartmental model for hepatobiliary transport of [^11^C]CSar with in vivo quantification of exchange rates of [^11^C]CSar between blood, hepatocytes and intrahepatic bile ducts inside the liver-VOI (Fig. 6)^4^. The transport kinetics are described by rate constants estimated by non-linear regression with *C*_in_(*t*) as input and *C*_liver_(*t*) as output functions. To reduce the number of fitting parameters, the *k*_2_/*k*_3_ ratio, i.e. the ratio between the rate of backflux and hepatobiliary secretion from the hepatocyte, was constrained to (*E*_0 _− *E*_AUC_)/*E*_AUC_^5^.

The average time that an [^11^C]CSar molecule resides in the hepatocyte before being either secreted into bile (*k*_3_) or back to blood (*k*_2_), *T*_hep_ (min), was calculated as:

$${T}_{hep}=\frac{1}{{k}_{2}+{k}_{3}}$$

#### Analysis of [^18^F]FDGal PET Data

The hepatic systemic clearance of [^18^F]FDGal, *K*_met_ (mL blood/min/mL liver tissue), was calculated voxel-by-voxel according to the Gjedde-Patlak model assuming irreversible trapping of [^18^F]FDGal^6,7^. The model was applied to data from 6 to 20 min after the [^18^F]FDGal injection using *C*_A_(*t*) as input, as previously described^30^.

### Statistical analysis

Data was analysed as two independent samples, and normality was assessed using Q–Q plots. All kinetic parameters from blood and PET data were normally distributed in both groups. Group values are accordingly expressed as group means and were compared using the two-sided Student’s *t* test (n = 5 per group; in analysis of [^11^C]CSar data, n = 4 in the irradiated group). Numbers of positive hepatocytes per 10 HPFs in the immunohistochemical stain were non-normally distributed. Group values are therefore expressed as group medians and were compared using the Wilcoxon rank sum test (n = 5 per group). Biochemical blood test values from irradiated pigs at baseline and after SBRT were compared using the Paired *t* test or Wilcoxon signed-rank test, when appropriate; baseline values from irradiated pigs and values from control pigs were compared as two independent groups (n = 5 per group). Correlations were estimated using the Person correlation coefficient. A *P* value below 0.05 was considered as statistically significant. Statistical analysis was performed using STATA (Version 14.2, StataCorp, College Station, TX) and SigmaPlot for Windows (Version 11.0 build 11.2.0.5).

## Data Availability

The datasets generated and analysed for the current study are available from the corresponding author on reasonable request.
